# Drug resistance in multiple myeloma: Soldiers and weapons in the bone marrow niche

**DOI:** 10.3389/fonc.2022.973836

**Published:** 2022-09-21

**Authors:** Antonio Giovanni Solimando, Eleonora Malerba, Patrizia Leone, Marcella Prete, Carolina Terragna, Michele Cavo, Vito Racanelli

**Affiliations:** ^1^ Department of Biomedical Sciences and Human Oncology, School of Medicine, ‘Aldo Moro’ University of Bari, Bari, Italy; ^2^ Istituto di ricovero e cura a carattere scientifico (IRCCS) Istituto Tumori ‘Giovanni Paolo II’ of Bari, Bari, Italy; ^3^ Department of Interdisciplinary Medicine, School of Medicine, ‘Aldo Moro’ University of Bari, Bari, Italy; ^4^ ’Seràgnoli’ Institute of Hematology, Bologna University School of Medicine, Bologna, Italy

**Keywords:** multiple myeloma, drug resistance, bone marrow microenvironment, monoclonal gammopathy of undetermined significance, therapeutic targets

## Abstract

Multiple myeloma (MM) is still an incurable disease, despite considerable improvements in treatment strategies, as resistance to most currently available agents is not uncommon. In this study, data on drug resistance in MM were analyzed and led to the following conclusions: resistance occurs *via* intrinsic and extrinsic mechanisms, including intraclonal heterogeneity, drug efflux pumps, alterations of drug targets, the inhibition of apoptosis, increased DNA repair and interactions with the bone marrow (BM) microenvironment, cell adhesion, and the release of soluble factors. Since MM involves the BM, interactions in the MM-BM microenvironment were examined as well, with a focus on the cross-talk between BM stromal cells (BMSCs), adipocytes, osteoclasts, osteoblasts, endothelial cells, and immune cells. Given the complex mechanisms that drive MM, next-generation treatment strategies that avoid drug resistance must target both the neoplastic clone and its non-malignant environment. Possible approaches based on recent evidence include: (i) proteasome and histone deacetylases inhibitors that not only target MM but also act on BMSCs and osteoclasts; (ii) novel peptide drug conjugates that target both the MM malignant clone and angiogenesis to unleash an effective anti-MM immune response. Finally, the role of cancer stem cells in MM is unknown but given their roles in the development of solid and hematological malignancies, cancer relapse, and drug resistance, their identification and description are of paramount importance for MM management.

## Introduction

Recent advances in the treatment of MM include the use of novel therapeutic strategies, such as immunomodulatory drugs (thalidomide, lenalidomide, pomalidomide), proteasome inhibitors (PIs: bortezomib, carfilzomib), monoclonal antibodies (MoAbs: daratumumab and elotuzumab), T-cell-based therapies, checkpoint inhibitors and hematopoietic stem cell transplantation. Nonetheless, MM remains an incurable disease (1–3), with 5-year survival rates ranging from 23.1% to 46.7%. For example, in the UK, survival has increased from 6% to 33%, but only 29% of patients will survive more than 10 years (4). Most patients suffer relapse because of the incomplete therapeutic eradication of malignant plasma cells (PCs). In MM, PCs can manifest drug resistance already before treatment or following conventional drug exposure (5). While patients initially respond to modern combination treatment regimens, they inevitably experience serial relapses, with the depth and duration of response following each relapse becoming progressively shorter until their disease becomes refractory (6). How drug-resistant PCs become dominant in MM and are able to persist despite multiple lines of therapy is poorly understood (7). However, elucidation of the mechanisms underlying drug resistance is a prerequisite for MM prevention and for effective therapies.

## Drug resistance mechanisms in MM

**Figure 1:**
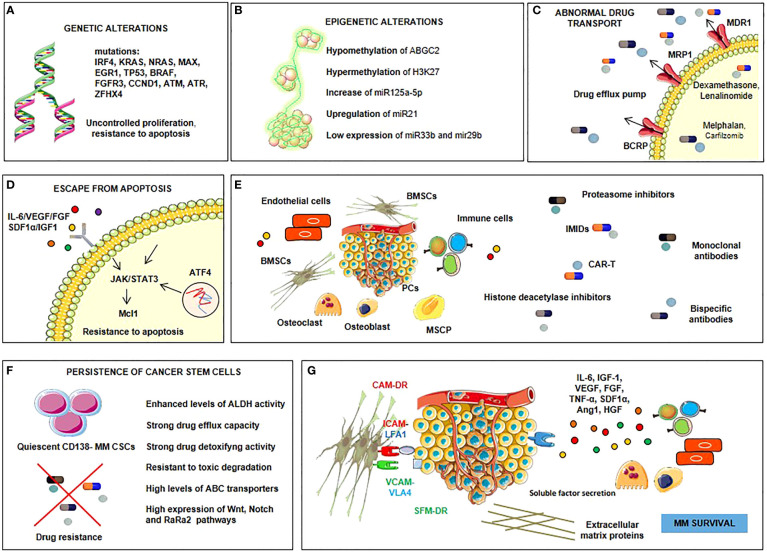
Drug resistance in MM involves intrinsic or extrinsic mechanisms. Intrinsic ones are genetic **(A)** and epigenetic **(B)** alterations. Genetic alterations **(A)** include mutations of a lot of genes such as interferon regulatory factor-4 (IRF-4), KRAS, NRAS, myc-associated factor X (MAX), early growth response protein 1 (EGR1), tumor protein p53 (TP53), BRAF, fibroblast growth factor receptor 3 (FGFR3), cyclin D1 (CCND1), ataxia telangiectasia mutated (ATM), ataxia telangiectasia and Rad3-related protein (ATR) and zinc finger homeobox 4 (ZFHX4), which procure uncontrolled proliferation and resistance to apoptosis. Epigenetic alterations **(B)** involve mechanisms of hypomethylation (ATP-binding cassette G2, ABGC2) and hypermethylation (histone 3 lysine 27, H3k27); upregulation of some microRNAs (miRNAs), such as miR125a-5p and miR21, and downregulation of others, such as miR33b and miR29b. Other intrinsic mechanisms are the overexpression of drug efflux pumps and the alteration of drug targets **(C)**, dysregulation of intracellular signaling pathways, such as those that mediate apoptosis (Janus kinase (JAK)-signal transducer and activator of transcription 3 (STAT3)-myeloid cell leukemia sequence 1 (Mcl1), autophagy (activating transcription factor 4, ATF4), and DNA repair **(D)**. Extrinsic mechanisms involve myeloma plasma cell interactions with the BM microenvironment **(E)**, persistence of cancer stem cells **(F)**, soluble factors-mediated drug resistance (SFM-DR) and cell adhesion-mediated drug resistance (CAM-DR) **(G)** resulting from the production of soluble factors such as interleukin (IL)-6, insulin-like growth factor-1 (IGF-1), vascular endothelial growth factor (VEGF), fibroblast growth factor (FGF), tumor necrosis factor-alpha (TNF-α), stromal cell-derived factor 1-alpha (SDF1-α), angiopoietin 1 (Ang1), hepatocyte growth factor (HGF) by bone marrow stromal cells (BMSCs) and plasma cells (PCs), and from the overexpression of cell cycle inhibitors, anti-apoptotic members of the B-cell lymphoma-2 (Bcl-2) family and ABC drug transporters in myeloma cells upon direct adhesion with BMSCs.

Drug resistance in MM can involve intrinsic or extrinsic mechanisms (**Figure 1**) (8, 9). The former includes genetic and epigenetic alterations, as occurs in other solid and hematological malignancies (10–14), the overexpression of drug efflux pumps (15), alteration of drug targets (16), and a dysregulation of intracellular signaling pathways, such as those that mediate apoptosis (17), autophagy, and DNA repair (18). Extrinsic mechanisms are mainly mediated by interactions with the bone marrow (BM) microenvironment (7, 19), cell adhesion to the extracellular matrix (ECM), and other elements of the tumor microenvironment (20), which may induce the production in MM cells of cell cycle inhibitors, anti-apoptotic members of the Bcl-2 family, and ABC drug transporters (21) (18, 22) as well as the release of soluble factors, such as interleukin (IL)-6 and insulin-like growth factor (IGF)-1, by bone marrow stromal cells (BMSCs) that activate the signal transduction pathways leading to drug resistance (23).

## The MM microenvironment

Myeloma cells reside in the BM, which forms a nutritional niche where they interact with BMSCs *via* CD44 and other adhesion molecules (24). A vicious cycle is established, driven by the secretion by BMSCs of factors that promote the proliferation of myeloma cells (IL-6, IGF-1, transforming growth factor (TGF)-β, and hepatocyte growth factor (HGF) (25–27). Moreover, due to the median age of disease onset, the BM is enriched with adipose tissue, which secretes tumor-promoting factors such as leptin and adiposin (28). One of the hallmarks of MM is the induction of bone lysis, the consequence of the secretion by malignant clones of osteoclast-activating factors such as MIP1-α and RANKL (29–31). In turn, osteoclasts secrete osteotropic factors, such as osteopontin and IL-6 (32), while the tumor mass produces the osteoblast inhibitors DKK1 and sFRP (33). The interaction of MM cells and osteoclasts is crucial; osteoclasts and MM plasma cells can recruit each other and mutually promote their survival through multiple mechanisms. To better understand these mechanisms, Moreaux et al. have analyzed the osteoclast-gene expression profiling and detected 552 genes overexpressed in osteoclasts compared with other BM cell subpopulations. They have identified genes coding for 4 CCR2-targeting chemokines that are expressed specifically by osteoclasts and genes coding for important MM growth factors such as IGF-1, IL-6, and a proliferation-inducing ligand (APRIL) (34, 35). This suggests that if plasma cell expresses CCR2, it could be attracted by osteoclasts specifically. Indeed, in the bone remodeling compartments CCR2 chemokines as well as plasma cell survival factors were highly concentrated. Furthermore, the bone lesion number in patients with newly diagnosed MM was associated with high CCR2 expression in myeloma plasma cells (34). Thus, targeting the osteoclast/MM plasma cell interaction through MoAb against CCR2 and/or MM growth factors may be a promising therapeutic approach. Importantly, *in vitro* experiments showed that an anti-CCR2 MoAb blocked osteoclast chemoattractant activity for myeloma cells, but it did not inhibit osteoclast MM cell growth activity. APRIL or IL-6 inhibitors specifically reduced osteoclast-induced MM survival to a partial extent. An anti-IGF-1 receptor MoAb totally suppressed the osteoclast-induced survival of MM plasma cells inhibiting both osteoclast and myeloma cell survival. The exact role of osteoclast in the BM milieu is not fully elucidated. However, the impact in terms of bone involvement prompted a plethora of preclinical and clinical studies aiming to target bone remodeling and, indirectly, tumor progression (29, 36, 37). All targets mentioned above could be potentially valuable in the next-generation immunotherapy for MM patients. For instance, a receptor for APRIL is the B-cell maturation antigen (BCMA), a novel encouraging target for MM immunotherapy. BCMA is a cell biomarker present mainly on CD138^pos^, CD38^pos^, CD45^neg^ cells, and it physiologically orchestrates plasma cell homeostasis (38). BCMA pathway highly depends on APRIL and BAFF, boosting MM cell pro-survival, and drug resistance (31, 39).

Furthermore, MM cells also induce neo-angiogenesis in the BM, through their release of the endothelial cell stimulators vascular endothelial growth factor (VEGF) and basic fibroblast growth factor (bFGF), which ensures the nutritional support of myeloma growth, including *via* stromal cell-derived factor (SDF)-1α, angiopoietin (Ang)-1, and HGF (40, 41). Based on these findings, the currently available treatment for MM targets both the malignant cells and the MM niche, through the inclusion of proteasome or HDAC inhibitors, which act on stromal cells, osteoclasts, and angiogenesis (42, 43).

## The immunological microenvironment in MM

The immunological microenvironment in patients with MM is unique, as are the genetics of the tumor itself. This was recently demonstrated by the use of high-content single-cell techniques (44). The observation that immune activation and exhaustion appear early in the pathogenesis of the preneoplastic phase termed monoclonal gammopathy of undetermined significance (MGUS) (44) suggests that features of the immune response necessary for the long-term stability and persistence of immunity, such as stem-like memory T cells, are critical determinants of immune control. Specific immune response targets may also be relevant, as it may be more effective to therapeutically target clonal rather than subclonal mutations or critical clone features such as stemness (36). Although the spatial elements of the immune response in hematologic malignancies have hardly been investigated, pathologists and radiologists have long recognized that myeloma is multifocal in its growth (hence the name multiple myeloma). Accordingly, understanding the spatial aspects of the immune response as well as the roles played by tissue-resident vs. recruited cells will be crucial in achieving immune control (37, 38).

The evolutionary and ecological context of the tumor must also be considered (39). The former refers to the heterogeneous genetic makeup of the tumor cells as well as changes therein over time. The ecological context is determined by immune cells and the availability of tumor-supporting resources, such as growth hormones and nutrients (39). In MGUS, the evolutionary path is largely established early in the disease, but it is subject to alterations over time. While it is thought that ecological factors, particularly those involving the immune system, are a major predictor of the tumor’s evolutionary trajectory, the respective interactions are likely to be regional. Consequently, the spatial elements of these interactions must be taken into account in the design of therapeutic agents, which must target both the MM cells and the immune niche; combinatorial techniques may thus be required for tumors with a greater rate of genetic change, but in other cases more conservative and sequential tactics may be more effective (36). The immune exhaustion that begins early in MGUS underscores the need to integrate immunologically based prevention with the early detection of the disease (37).

Moreover, the myeloma niche is immunosuppressive, as the activity of myeloid-derived suppressor cells (MDSCs), which secrete IL-10 and TGF-β (45), is enhanced by the production of IL-6, and granulocyte-macrophage colony-stimulating factor (GM-CSF) by MM cells. MDSCs also inhibit T-cell function, by releasing arginase and inducible nitric oxide synthase, and stimulate neo-angiogenesis by releasing VEGF (46–48).

## The role of mesenchymal stem cells in MM

De Jong et al. presented a single-cell transcriptomic analysis of the hematopoietic and non-hematopoietic BM microenvironment in MM and identified myeloma-specific BM iMSCs able to create an ideal environment for tumor cell proliferation and immune cell recruitment and modulation (49). These cells produced a wide range of inflammatory cytokines and chemokines, such as IL-6, COX2, CXCL2, CXCL8, VEGFA, proteins in the tumor necrosis factor (TNF) pathway, and CCL2, which increases MM cell motility through its association with CCR2 (49). These populations also expressed genes encoding CXCL3, CXCL5, and CD44, the latter of which was proposed as a flow cytometry marker of iMSC, despite the fact that it is not expressed by all iMSCs (50). De Jong et al. identified the expression of IL-1 and TNF receptors on MSCs as determinants of the iMSC phenotype. This was confirmed by the ex vivo activation of MSCs with IL-1 and TNF, which resulted in the iMSC phenotype in both healthy and MM-derived MSCs. The authors examined the interactions between MSC receptors and immune and tumor cells and found that IL-1B was largely expressed by monocytes, and TNF by cytotoxic T and NK cells. In addition to interacting with myeloid cells, iMSCs engaged with proliferating myeloma cells through the CCL2–CCR2 pathway. Interestingly, De Jong et al. revealed an immune cell-mediated stromal inflammation in BM of MM patients persisting even after successful induction therapy, suggesting a potential role of iMSCs in disease relapse (49).

## The role of hypoxia in MM and its treatment

Novel candidate targets that can be used to overcome drug resistance in MM have been identified by dividing the BM microenvironment into the low-oxygen endosteal niche and the high-oxygen vascular niche (51). MM cells reside in the endosteal niche, where the hypoxic environment induces changes in the availability of metabolites, such as amino acids, fatty acids, and nucleotides (52), and induces the enhanced shedding of exosomes (discussed below, 53), both of which contribute to the drug-resistant phenotype (54). Among the significant changes in myeloma cell metabolism in response to hypoxia (55, 56) are those affecting the levels of hexokinase-2 (HK-2) and lactate dehydrogenase A (LDHA, 56). Both have been linked to resistance to bortezomib, a PI shown in an *in vivo* model to decrease myeloma growth (55, 56). Ikeda et al. focused on KDM3A, another target of hypoxia in myeloma, and found that hypoxia caused an up-regulation of KDM3A that was paralleled by the up-regulation of the downstream protein MALAT1. Thus, according to this result, KDM3A induces MALAT1, which, in turn, induces glycolysis, leading to the proliferation of MM cells (55).

Proteomic profiling was used to examine the changes induced in myeloma cells obtained from patients with active disease. Janker et al. compared cells obtained from healthy donors to MM cells from patients with either a low or a high tumor burden (57). They found that most of the metabolic enzymes were modified in response to hypoxia, thus suggesting that most of the metabolic alterations in MM can be explained by the low oxygen concentration (57). However, studies of agents directed at hypoxia-sensitive targets, such HK2 inhibitors (58), the LDHA-specific inhibitor GSK 2837808A (59), demethylase KDM inhibitors (60), and antisense oligonucleotides (60), are at the preclinical stage. As a proof of concept, Xu et al. used an antisense oligonucleotide against HK2 in a xenograft model of an OPM-2 tumor and reported decreased MM progression (58).

## Exosomes as a therapeutic target in MM

Exosomes are small (100 nm) vesicles that are secreted by most cell types from multivesicular bodies and taken up by endocytosis or micropinocytosis. Importantly, however, they are involved in information transfer, including within the BM environment (31). MM-derived exosomes induce angiogenesis (53, 61), are involved in osteoblast functionality (62, 63) and boost cancer-associated fibroblasts activity in the MM niche (64–66). Roccaro et al. found that exosomes from MSCs of the BM of MM patients stimulate and those from healthy donors reduce the proliferation of myeloma cells, suggesting MM stromal cells as druggable targets (67). These observations were paralleled by *in vivo* results obtained using silk scaffolds (67). The effect of MSC-derived exosomes in bortezomib resistance was also investigated, with exosomes treatment shown to reduce proteasome inhibition (68). In a proteomic profiling study, Wang et al. compared exosomes from MM cells with those from stromal cells and found the up-regulation of MCP1 and SDF1 in the latter. Furthermore, exosomes from both were shown to be enriched in IL-6 and fibronectin, and exosomal miR15a was shown to be involved in MM suppression (67).

A comparison of the exosomes from the BM MSCs of bortezomib-resistant vs. bortezomib-sensitive MM patients revealed significantly higher levels of PSMA3-AS1, encoding a subunit of the proteasome, in resistant patients (69). Sensitivity could be restored by PSMA3 antisense treatment. These results were corroborated by an *in vivo* model in which MM mice treated with carfilzomib and PSMA-silencing RNA had reduced tumor development and improved survival (69). Studies of drug-exposure-dependent exosome secretion have shown the increased secretion of exosomes by MM cells treated with melphalan, bortezomib, or carfilzomib, with the respective chemoexosomes containing more heparinase on the surface and enriched with cell cycle proteins (70). The chemoexosomes of MM cells treated with bortezomib and melphalan were also shown to contain acid sphingomyelinase (ASM), which converts sphingomyelin to ceramide (71). The induction of drug resistance by treating bortezomib-sensitive JJN3 cells with bortezomib-resistant U266-cell-derived exosomes suggested that blocking ASM could restore drug sensitivity. Blocking exosome release, such as with the exosome inhibitor GW4869, to modulate osteolysis has been proposed as well (71). In that study, mice were treated with GW4869 in combination with bortezomib (71). Whereas GW4869 alone did not affect MM burden it synergized with bortezomib, whereas with respect to osteolysis GW4869 was effective as a single agent, with its use resulting in enhanced cortico-bone volume and decreased serum collagenase levels (62).

Collectively, exosomes link MM to metabolism and drug resistance, since they contain small molecules and miRNA/lncRNA, inducing metabolic changes in their target cells. They also contain metabolites (such as lactate), metabolic enzymes, and transporters (72). In MM, hypoxia induces changes in metabolism and exosome secretion, which in turn influence MM growth. Taken together, these observations point to exosomes as novel targets to overcome drug resistance.

## Therapeutic targeting of the tumor microenvironment in MM

**Figure 2:**
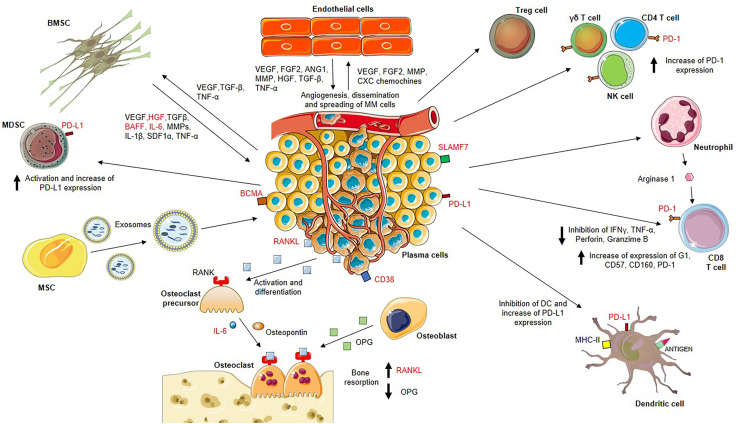
MM BM microenvironment is highly enriched in factors that sustain the proliferation of bone marrow-resident cells including myeloma plasma cells, endothelial cells and bone marrow stromal cells (BMSCs), actors of two important vicious cycles. On one hand, BMSCs release vascular endothelial growth factor (VEGF), hepatocyte growth factor (HGF), transforming growth factor-β (TGF-β), B cell activating factor (BAFF), interleukin (IL)-6, matrix metalloproteinases (MMPs), IL-1β, stromal cell-derived factor 1 alpha (SDF1α), tumor necrosis factor-α (TNF-α) which stimulate myeloma plasma cell proliferation. On the other hand, myeloma plasma cells produce VEGF, TGF-β, TNF-α sustaining BMSC growth and activation, and endothelial cell stimulators such as VEGF, FGF2, MMP, CXC chemokines promoting neo-angiogenesis. Stimulated endothelial cells also release VEGF, FGF2, angiopoietin 1 (ANG1), MMP, HGF, TGF-β, TNF-α which ensure the nutritional support for myeloma growth. Moreover, MM BM mesenchymal stem cells (MSCs) release exosomes with higher expression of oncogenic proteins, cytokines, protein kinases and microRNAs (miRNAs) that are transferred to myeloma plasma cells resulting in MM growth. Disease progression is also promoted by the strongly immunosuppressive BM microenvironment. MM plasma cells induce activation of T regulatory (Treg) cells and myeloid-derived suppressor cells (MDSCs), and increase of the programmed cell death-1 (PD-1) expression on gamma delta (γδ) T cells, CD4 T cells, natural killer (NK) cells and CD8 T cells. Neutrophils secrete arginase 1 involved in the dysfunction of CD8 T cells due to increased expression of PD-1, G1, CD57, CD160 and decreased secretion of IFNγ, TNF-α, perforin and granzyme (B) Dendritic cells also undergo inactivation and increase levels of inhibitory markers such as programmed death-ligand 1 (PD-L1).

In MM, the tumor microenvironment induces angiogenesis and hypoxia and alters the ECM as well as MSCs, osteoclasts, osteoblasts, and immune cells (73), representing promising druggable targets as stand-alone or in combinatorial approaches as demonstrated in solid and hematological cancers (74, 75). For example, the co-culture of MM cells with MM MSCs leads to enhanced tumor cell proliferation and adhesion. The gene-expression profile of MSCs from MM patients differs significantly from that of MSCs obtained from patients with multiple myeloma (76). Several vicious cycles are operative within the MM niche since MM cells make use of adhesion molecules and paracrine loops to interact with endothelial cells. Endothelial-multiple myeloma interactions feed into redundant cross-talk, propagating disease progression (77) (22, 27, 78–79) (**Figure 2**).

Myeloma plasma cells express and release the receptor activator of nuclear factor kappa beta ligand (RANKL) that binding to its receptor RANK on osteoclast precursors promoting their differentiation into bone-resorbing osteoclasts. Activated osteoclasts secrete osteotropic factors including osteopontin and IL-6, which support bone resorption. The increased osteoclast activity results from the enhanced RANKL production and the reduced osteoprotegerin (OPG) production by osteoblasts.

Potential drug targets are marked in red.

Drug-based attempts to break the lines of communication between MM cells and their environment have demonstrated clinical efficacy, including in overcoming resistance (69, 80) (**Table 1**). A related strategy is to alter the tumor milieu through druggable targets such as CXCR4, IL-6, IGF-1, VEGF, their cognate receptors, and RANKL (81) in combination therapy to target the cytokines involved in cell adhesion (82) has shown promise whereas monotherapy has been ineffective.

**Table 1:** Phase III-IV clinical trial aiming to overcome drug resistance in MM, targeting bone marrow microenvironment.

TARGET	CLINICAL TRIAL	PHASE
**BCMA**	NCT04923893	III
	NCT04287660	III
	NCT04181827	III
	NCT05020236	III
	NCT05317416	III
**SLAMF7**	NCT03948035	III
	NCT01891643	III
**CD38**	NCT02419118	III
	NCT04270409	III
	NCT03275285	III
	NCT02990338	III
	NCT02990338	III
	NCT03319667	III
	NCT05020236	III
	NCT04751877	III
	NCT01891643	III
	NCT03937635	III
	NCT04246047	III
	NCT04566328	III
**PD-1**	NCT03357952	II/III
	NCT02579863	III
	NCT02576977	III
**PD-L1**	NCT02576977	III
	NCT02579863	III
**RANKL**	NCT01045460	III
	NCT02943473	III
	NCT02471820	III
**HGF**	NCT01100879	IV
**IL-6**	NCT01266811	III
	NCT01100879	IV

Among the novel anti-MM agents that target the MM niche are alkylators, HDAC inhibitors, PIs, and MEK/ERK and Bcl-2/Mcl-1 inhibitors (83). Alkylators are of interest as they induce tumor cell lysis as well as a more immunogenic niche, by sensitizing MM cells to other immunotherapeutic approaches such as MoAbs. Novel alkylators include melphalan flefenamide (melflufen), which causes the formation of a radical that makes the alkylator inactive and lipophilic. Once inside the cell, the radical is released by the actions of aminopeptidases, which are highly enriched in MM cells, such that high intracellular concentrations of melphalan are obtained (84–87). In the Horizon trial (OP-106) (88), the overall response rate to melflufen was 29%, with the drug shown to also be active in patients with triple refractory disease and those with extramedullary disease. The benefits of an alkylator that may also act in the immune system was evidenced by the ~4-month increase in progression-free survival (PFS). The combination of melflufen with daratumumab and dexamethasone (NCT03481556) may sensitize patients to these MoAbs. Similarly, the alkylator DAC has several immunological effects, involving CD8, NK cells, and MDSCs (89, 90). EDO-S101 is a bendamustine derivative that combines an alkylator with the HDAC inhibitor vorinostat. The latter opens the chromatin, boosting the alkylator’s efficacy. EDO-S101 induces potent DNA damage in MM and may synergize with daratumumab (91).

Kroenke elucidated the mechanism of lenalidomide activity in MM (17, 92) by identifying cereblon (CRBN) as a putative target of the drug and demonstrating that the binding of CRBN to the transcription factors IKZF1 (IKAROS) and IKZF3 (AIOLOS) leads to their proteasome-mediated degradation. The destruction of these factors accounts for lenalidomide’s cytotoxic effect, at it causes the down-regulation of IRF4 and Myc, resulting in an immunomodulating effect that includes an increase in IL-2 and a decrease in TNF (92). Whether IKZF1 and IKZF3 are predictive markers for immunomodulatory drug (IMiD) therapy is thus far unclear (93). In addition to inhibiting the IKAROS-mediated gene expression program (94), lenalidomide inhibits a CRBN-mediated E3 ligase, thus demonstrating pleiotropic, clinically relevant effects in immunomodulation (93, 95). Nonetheless, lenalidomide resistance has been traced to mutations in the CRBN binding region of IKZF1 and IKZF3 (96). This observation prompted intensive research focusing on novel compounds such as iberdomide (CC-220), shown to elicit a 30% response, also in heavily pretreated patients (97), and CC-92480, which is also active against extramedullary disease (98). The biological role of the RAF/RAS pathway in the BM microenvironment (99) led to investigations into the role of RAS in MM. In 2014, Lohr et al. showed that genes encoding RAS family members are the most frequently mutated genes in MM (100), which raised interest in BRAF/MEK targeting (101–104).

Venetoclax is a potent, selective, orally bioavailable, small molecule that inhibits the apoptosis regulator BCL-2 (discussed in detail below). It holds great promise in MM based on its induction of cell death in MM cell lines and primary tumor samples, especially those with t (11, 14) translocation, in which an increased dependency upon BCL-2 for MM cell survival has been determined (105–107). Modulation of the anti-apoptotic pathway by venetoclax, by enhancing the binding of MM cells to the microenvironment, has been proposed (108), and the drug’s activity in t (11, 14) MM (109) and in combination with bortezomib and dexamethasone has been described. In another study, venetoclax improved PFS in patients with t (11, 14) or BCL2^high^ MM, but not in patients with non-t (11, 14) BCL2^low^ MM (110). The finding that MCL-1, an inhibitor of apoptosis, and BCL-2 levels are modulated by the tumor-associated microenvironment (111) has stimulated further investigation focused on MCL-1 inhibition.

Another target in the tumor microenvironment is arginine, which is required for the activation and proliferation of T lymphocytes and NK cells (112) while exerting an immunosuppressive effect. MDSCs and neutrophils produce high levels of arginase (113), which has led to exploration of the therapeutic potential of the arginase inhibitor INCB001158 in combination with daratumumab. Phase I/II clinical trials are being conducted.

Finally, several of the strategies and agents used in the treatment of non-Hodgkin lymphomas (83, 114), such as IMiDs, CELMoDs, naked MoAbs (anti-CD38 or SLAMF-7), ADCs, CAR-Ts, and BiTEs, are state-of-the-art tools being tested for their ability to overcome MM drug-resistance.

## Escape from apoptosis and other mechanisms of drug resistance

An important hallmark of cancer is the evasion of apoptosis. This pathway, which is under the control of BCL-2 family proteins, therefore represents an attractive therapeutic target (115). Programmed cell death is initiated by signaling processes, including the NF-kB, PI3K/AKT, and proteasome pathways (116). IL-6, VEGF, fibroblast growth factor (FGF), SDF1α, and IGF-1 trigger the MAPK/ERK and the JAK/STAT 3 pathways, which have a crucial role in the induction of apoptosis (117–119). Sequestering both BH3 and effector pro-apoptotic proteins blocks apoptosis, as a mechanism of drug resistance that might be overcome using BH3-mimetics. These include the above-discussed BCL-2 inhibitor venetoclax and S64315, AMG176 AZD5991 which target MCL-1 (115). The overexpression of anti-apoptotic BCL-2 proteins accounts for the vulnerability of MM cells to BH3- mimetics. As noted above, venetoclax induces a large response in t (11, 14) MM patients, and specifically those with low-level expression of BCL-XL and MCL-1 resistance factors (109). BH3-mimetics have demonstrated that the many cell lines require MCL-1 for their survival, and MCL-1 dependency was shown to increase in MM relapse (120). Mitsiates et al., demonstrated that TNF–related apoptosis-inducing ligand/Apo2 ligand (TRAIL/Apo2L) causes apoptosis in human MM cells, MM cell lines, and the MM cells of patients with disease either sensitive or resistant to dexamethasone (Dex) and chemotherapy. Doxorubicin and NF-Kβ inhibitors increase TRAIL/Apo2L expression in MM cells, but not in normal B cells, which suggests the targeting of TRAIL/Apo2L as a novel therapeutic strategy in MM (104).

NF-kB family members regulate growth, cell differentiation, and apoptosis in cell lines and tissues but they also play a role in drug resistance and in MM pathogenesis. Studies using the IKK inhibitor PS-1145 have confirmed the role of NF-kB in the growth, survival, and drug resistance of MM cells and therefore raised interest in the targeting of NF-kB in MM (121). Qu et al. found lower NF-kB expression in drug-sensitive than in drug-resistant cells and the up-regulation of NF-kB in the MM cells of patients with disease relapse. The authors showed that NF-kB could be blocked using arsenic trioxide, bortezomib, or Ikβ kinase inhibitors, all of which induced apoptosis in MM cell lines (122).

Activation of the unfolded protein response (UPR) pathway decreases ER stress, inhibits protein synthesis, and increases the transcription of heat shock proteins (HSP) that act as folding chaperones (123, 124). Misfolded proteins are also degraded by proteasomes and autophagy (125). MM cells are characterized by high levels of misfolded or unfolded proteins in the endoplasmic reticulum (ER), which causes their strong dependence on the UPR pathway for survival. Accordingly, MM cells are highly sensitive to PIs such as bortezomib, which inhibits proteasome activity and results in increased levels of misfolded protein in the ER, in turn inducing apoptosis in malignant cells (126). Gambella et al., demonstrated a correlation between higher levels of X-box binding protein 1 (XBP1) and a higher sensitivity to bortezomib (104). Nikesitch et al., showed correlations between ER size, decreased levels of the UPR regulator ATF6, the reduced expression of XBP1 activator, and bortezomib resistance. These observations suggest that decreased UPR activity predicts PI resistance (123).

Another essential survival mechanism for MM cells is autophagy, which helps malignant cells to degrade misfolded proteins and is associated with drug resistance. Milani et al. showed a correlation between the up-regulation of autophagy-inducer activating transcription factor 4 (ATF4) and bortezomib resistance (127). Autophagy thus represents another therapeutic target in MM (128, 129). Indeed, the combination of autophagy inhibitors and bortezomib has been tested in phase I and II clinical trials, with promising results obtained in patients with relapsed refractory disease (130). Carfilzomib in combination with autophagy inhibitors has also shown success in increasing apoptosis levels, both *in vitro* and *in vivo* (131).

The heat shock proteins HSP70 and HSP90 play a role in MM survival (132). HSP90 ensures the stability of anti-apoptotic signaling proteins, such as AKT, STAT3, and IL-6 receptors (133). A combination of inhibitors of HSP90 and other drugs has been examined for their ability to activate apoptosis, both *in vitro* and *in vivo* (134, 135).

Shadel et al. demonstrated that, in response to chemical stress, mitochondria activate molecular alarm signal triggered by nuclear DNA (nDNA) damage. Previous evidence showed that the response of cells to mitochondrial DNA (mtDNA) changes was similar to the response to an external pathogen, as in both cases, mediators capable of activating cell protection programs are released (136). The authors examined several anti-tumoral drugs, such as doxorubicin, and showed that they damage mtDNA and nDNA, causing the expression of a specific subset of interferon-stimulated genes (ISG), which are usually activated by a virus. The ISGs, including Parp9, remain activated by the unphosphorylated form of ISGF3 (U-ISGF3), which enhances nDNA damage and repair responses and facilitates chemoresistance. mtDNA is therefore a good genotoxic stress sentinel, given its higher sensitivity to the absence of repair (137).

The Bcl-2 inhibitor venetoclax, an inhibitor of BCL2, and compound C, an inhibitor of AMP-activated protein kinase, are more effective in bortezomib and carfilzomib resistant cells, due to their targeting of mitochondria. Moreover, it has been observed a switch in lipid class from lysolipids to sphingomyelins with the accumulation of mono-acylglycerols in PI-resistant cells. A comparison of D609, an inhibitor of sphingomyelin synthase and the sphingomyelin transferase inhibitor tamoxifen on PI-resistant and PI-sensitive cells showed the greater cytotoxicity of D609 in PI-resistant cells than PI-sensitive cells, with the opposite effect determined for tamoxifen. These results suggest the importance of sphingolipid synthesis in PI resistance. The combination of D609 and bortezomib or carfilzomib exhibited synergistic effects in primary MM cells (138). Together, these findings encouraged the study of new compounds that induce cellular oxidative damage or mitochondrial damage. Some of these drugs had acceptable safety profiles but nonetheless promoted the killing of PCs and therefore hold promise as adjuvants in the treatment of MM (139). Song et al., showed that 2-methoxyestradiol increases human MM cell death, by stimulating the production of mitochondrial ROS and raising Ca^2+^ levels within the cells, due to the increased activation of c-Jun N terminal kinase (JNK) and mitogen-activated protein kinase 4/7 (MKK4/7, 140). Papanikolaou et al. demonstrated the mechanism by which artesunate (ART), a well-tolerated anti-malarial drug, induces apoptosis of MM cell lines by targeting the mitochondria and increasing mitochondrial membrane permeability. In response, the cytosolic and subsequent nuclear translocation of the mitochondrial proteins apoptosis-inducing factor (AIF) and endonuclease G (EndoG) is induced. The nuclear translocation of AIF and EndoG is accompanied by low ROS levels and the increased mitochondrial production of superoxide.

A further target of ART in cancer cells is heme, as the drug reduces heme (bivalent iron) to hemin (trivalent iron). Heme is essential to many protein complexes, such as cytochrome C, a component of the electron transport chain of mitochondria (141). Accordingly, the mitochondria, with their abundance of iron, are an ideal target of ART (141). A recent study showed that the activation of TLR4 signaling in MM cells promotes mitochondrial biogenesis and thus resistance to BTZ. The authors found that TLR4 activation by LPS stimulation increases MM cell mitochondrial mass and enhances the expression of PGC1α, a regulator of mitogenesis, of PRC, a transcriptional cofactor activating genes involved in mitochondrial respiratory function, and of TFAM, a regulator of mitochondrial biogenesis that promotes mtDNA replication and transcription. Tests of the TLR4 inhibitor TAK-242 in combination with bortezomib in human MM cell lines showed higher oxidative stress, due to increases in reactive oxygen and nitrogen species, which in turn caused mitochondrial membrane depolarization and cytochrome c release into the cytosol. This was followed by caspase-9 activation, resulting finally in the overcoming of MM cell resistance (142).

In another study, the authors tested the biological effects of the immunomodulator FTY720 on MM cells and demonstrated potent cytotoxicity against drug-sensitive and drug-resistant MM cell lines as well as against the tumor cells of MM patients whose disease did not respond to conventional drugs. FTY720 triggers the activation of caspase-8, -9 and -3 and causes poly(ADP-ribose) polymerase cleavage. Moreover, it alters mitochondrial membrane potential and causes Bax cleavage, which is followed by the translocation of cytochrome c and Smac/Diablo from the mitochondria to the cytosol, where they activate apoptosis. The addition of FTY720 to the combination of Dex and anti-Fas antibodies improved the response rate and overcame acquired drug resistance (143). These studies show that a reliance solely on diagnostic definitions and the criterion of MRD as the best prognostic marker are insufficient. Rather, investigations of specific compounds and their mechanisms will allow a patient-tailored approach to the treatment of MM (139).

## The persistence of cancer stem cells

Relapse and tumor progression are mainly caused by a rare population of cancer stem cells (CSCs), which survive treatment and give rise to a PC tumor (144). The characteristics of CSCs include immaturity, quiescence, embryogenic gene expression, drug resistance, and self-renewal (145). MM CSCs differ from normal stem cells in terms of their genetic and epigenetic features, with their phenotype determined by the nature of the mutations (145). In MM, the phenotype of CSCs is not precisely known (146), with some studies claiming that they resemble CD138− B cells (9, 147). Human MM cell lines were shown to contain small subpopulations of CD138− cells with greater clonogenic potential than the corresponding CD138+ cells. CD138− cells were also shown to exhibit stem cell properties, such as enhanced aldehyde dehydrogenase (ALDH) activity (**Figure 1F**), and were clonogenic both *in vitro* and *in vivo*, unlike CD138+ cells. The CD138− PC phenotype includes markers of normal B cells (CD19, CD20 and CD27), suggesting that MM CSCs arise from populations of clonotypic B-cells (148). Another characteristic of CSCs is their resistance to Dex, bortezomib, and lenalidomide, whereas the same drugs inhibit the growth of CD138+ PCs (146). Brandl et al. found a higher proportion of CD138+ cells in proliferating and engrafted cells, and a lower proportion or the absence of CD138 low/CD138− cells, shown to be highly positive for the cell-adhesion molecule JAM-C (junctional adhesion molecule-C, 147). More motile and thus faster disseminating. MM cells are mostly CD138+ at the time of MM diagnosis, but a remarkable percentage of tumor cells lose CD138 expression at relapse. Nonetheless, Kim et al., demonstrated that clonotypic CD138+ PCs share some qualities with CSCs such as self-renewal, tumor-initiating potential, and drug resistance (149). Gao et al., conducted gene expression profiling of CSCs and of MM cells derived from 11 MM patients and found that CD24+ MM cells displayed features of CSCs, including self-renewal and drug resistance, suggesting these cells as a target for MM treatment (9, 146).

Due to their strong drug efflux capacity and drug detoxifying activities, MM CSCs are highly drug-resistant. They also express higher levels of ABC transporters and ATP-binding cassette transporters. These transmembrane proteins use the energy of ATP hydrolysis to transport harmful chemicals across the membrane. One of the key causes of multidrug resistance and chemotherapeutic failure in MM is the ABC transporter-mediated active efflux of drugs (148). The ABCG2 transporter is overexpressed in MM side population (SP) cells, a small subset of tumor cells that can efflux the fluorescent DNA-binding dye Hoechst 33342. ABCG2 inhibition with cyclosporine analogues or verapamil reduces the fraction of CSCs, in turn re-sensitizing MM cells to vincristine, doxorubicin, and Dex (150). A comparison of the expression of ABC transporters in SP cells with that in the main population cells of human MM cell lines showed that ABCG2 expression was significantly higher in SP cells, except in doxorubicin-resistant RPMI-Dox40 SP cells and SP cells of the KMS-11 cell line, which expressed very high levels of ABCB1 (151). SP cells exhibit CSC-like characteristics, such as tumorigenic potential, stem-like gene expression, dye extrusion capacity, and chemoresistance due to the enhanced expression of membrane-bound drug transporters (152). Although previous research suggested that MM SP cells were highly clonogenic and contained CD138− but not CD138+ PCs (148), new evidence suggests that MM SP cells contain both CD138+ and CD138− populations (151). This finding implies that malignant PCs can acquire the SP phenotype, which has consequences for their treatment resistance.

Moreover, contact with BMSCs increases both the number of SP cells and their ability to proliferate. Drugs that target BMSCs and block this stimulatory impact, such as lenalidomide and thalidomide, reduce the percentage of SP cells considerably (151). Both lenalidomide and thalidomide strongly reduce the percentage of SPs, underlining the relevance of the BM microenvironment to cell growth and phenotype acquisition (151).

In addition to ABC transporters, ALDH, a member of the NAD(P)1-dependent enzymes family involved in the metabolic detoxification of aldehydes, including ethanol and cytostatic medications, is another major mediator of drug resistance in MM CSCs (153). ALDH is expressed at high levels in both normal cells and CSCs (154). ALDH1 is also expressed by MM cells that have increased proliferation and tumorigenic features and chromosomal instability linked to treatment resistance (155). Matsui et al. (148) found that ALDH was upregulated in CD138− cells.

In their dynamic and context-dependent phenotypic reprogramming, MM CSCs, like other CSCs, use a variety of pathways, including Wingless (Wnt), Hedgehog (Hh), Notch signaling, and PI3K/Akt/mTOR (156–159). The Wnt pathway promotes cell proliferation, migration, invasion, drug resistance, and the creation of CSCs in MM (154). The expression of Wnt signaling pathway genes is increased in MM cells (160). Peacock et al. (2007) showed that Hh signaling is involved in keeping MM CSCs in an undifferentiated state, allowing clonal expansion. Hh activity is concentrated in CD138−/CD19+ MM CSCs but not in CD138+/CD19- MM PCs, demonstrating that stromal-derived Hh ligands promote MM CSC proliferation without differentiation (161).In CD138+ plasma cells and MM CSCs, overexpression of the retinoic acid receptor alpha 2 (RAR2), results in abnormal Wnt and Hh pathway activation and increased cell self-renewal, proliferation, migration, and drug resistance (160, 162). High-level Notch expression was demonstrated in clonotypic B cells from the BM of MM patients, suggesting that Notch is involved in maintaining MM CSC (163).

MiRNAs have emerged as major CSC regulators, with a distinct expression profile in CSCs and non-CSCs (164). A comparison of SP (side population) cells from MM cell lines and primary MM tumor cells identified ten miRNAs expressed at higher levels and 33 at lower levels. Among the miRNAs in SP cells, miRNA-451 was detected at high levels and shown to play a role in PI3K/Akt/mTOR pathway activation (146).

Contact with BMSCs boosted the number of SP cells and their ability to proliferate. Furthermore, drugs that target BMSCs and block this stimulatory impact, such as lenalidomide and thalidomide, reduced the percentage of SP cells considerably (151). Both lenalidomide and thalidomide strongly reduced the percentage of SP cells when they were co-cultured with BMSCs, underlining the relevance of the BM microenvironment (151).

The identification of more immature pre-plasmablastic cells has been uncovered to be relevant in specific scenarios, such as proteasome inhibitor resistance in MM. According to Orlowski et al., examination of patient samples identified up to five distinct subpopulations of tumor cells, including B-cells, activated B-cells, pre-plasmablasts, plasmablasts, and plasma cells, each of which may respond differently to bortezomib (165). B-cell and pre-plasmablast progenitors were discovered to survive proteasome inhibition and to be highly concentrated in samples resistant to bortezomib, supporting this theory. These results have significant implications for both innate and acquired proteasome inhibitor resistance. First, the authors claim that presumably all patients already have the potential for bortezomib resistance and cross-resistance with other PIs and that this disease phenotype will eventually emerge in step with the loss of bortezomib-sensitive plasmablasts and plasma cells (165). Thus, the identification of the baseline amount of these progenitor cells by whole genome sequencing or gene expression profiling may predict the PIs sensitivity (165). Therefore, promising therapeutic window can be directed to both committed plasma cells and progenitor, or stem cell-like cells. More interestingly, as Leung-Hagesteijn et al. corroborated the insights regarding the MM progenitors by describing a disease’s architecture that mimics the stages of B cell and plasma cell maturation and is a determinant in clinical PI resistance. While maturation defect before the plasmablast stage allows progressive disease on PI treatment in MM, Xbp1s tumor B cells and pre-plasmablasts can be resistant to PI. Indeed, bortezomib resistance is also developed through spliced X-box binding protein 1 (Xbp1) suppression, which reduces immunoglobulin production and consequently endoplasmic reticulum (ER) stress and susceptibility to PI (166). These findings emphasize the tumor progenitor makeup in MM and its contribution to disease progression and drug resistance (167).

Although stem cell properties are limited to a small percentage (possibly < 1%) of the tumor cell population, their eradication along with the bulk of tumor cells is likely to be crucial in obtaining a stable and durable remission, and possibly a cure for MM (9, 146).

## Epigenetic makeup and drug resistance in MM

Epigenetic modifications regulate normal B-cell development and plasma cell differentiation, and several epigenetic changes are implicated in myeloma pathogenesis and drug resistance (168–170). Alaterre E et al. extended this knowledge by examining the association between treatment response and the differential enrichment of H3K4me3 (an epigenetic modification of the histone H3) on gene promoters in Human multiple myeloma cell lines (HMCLs). Based on this histone alteration, the authors created an epigenetic biomarker predicting HMCL responses to lenalidomide and romidepsin (171). As mentioned earlier, the cereblon complex is a target of IMiDs. In the presence of IMiDs, Cereblon, composed of CUL4, RBX1, DDB1, and CRBN proteins, stimulates the ubiquitination of the B-cell transcription factors IKZF1 and IKZF3 (171, 172). A remarkable difference between lenalidomide-sensitive and -resistant HMCLs in step with H3K4me3 enrichment on the promoter of CUL4B protein was also described, establishing a link between the CUL4B splicing variation 1 and lenalidomide and pomalidomide sensitivity (171).

PIs sensitivity makes no exception. Indeed, with different approaches, De Smetd et al. characterized the role of G9a/GLP complex as a promising epigenetic regulator in MM; the authors showed that G9a/GLP targeting in MM cells induce autophagy-associated apoptosis and makes MM cells more susceptible to PI-based therapy by blocking mTOR signaling and lowering c-MYC levels. Therefore, PI-based therapy for patients with G9a high myeloma can be improved by G9a/GLP targeting (173).

Furthermore, key epigenetic factors that may be possible targets in myeloma include proteins well-known to be crucial in myeloproliferative/myelodysplastic disorders such as IDH2, DNMT3A/B, MMSET (WHSC1/NSD2), SETD2 and the polycomb repressive complex 2 (PRC2) complex (EZH2/PHF19); nonetheless, they are also promising target in MM. From this standpoint, PRC2 core genes were shown to be significantly upregulated in MM cells (174). Herviou et al. tested the impact of EPZ-6438, a selective small molecule inhibitor of EZH2 methyltransferase activity, on the phenotypic and gene expression profile of MM cells. Cell cycle arrest and cell death seem to be impacted due to the activity of polycomb and DNA methylation target genes. DNA methylation of PRC2 target genes mediates resistance to EZH2 inhibitor (171, 174). These results also provide the basis for a synergistic effect with lenalidomide and epigenetic modulators. Of note, in MM cells resistant to EZH2 inhibitor, Herviou L et al. pinpointed a considerable overlap between H3K27me3 and DNA methylation of EPZ-6438 target genes. There is a pathobiological connection between these two epigenetic repression systems. In fact, the PRC2 complex subunit EZH2 is necessary for the methylation of its target gene promoter. These results suggest that PRC2 target genes include essential MM tumor suppressor genes that various epigenetic pathways have repressed. Consequently, the therapeutic potential, since sublethal dosages of DNMTi can make EPZ-6438-resistant MM cell lines sensitive to EZH2 inhibitor (175, 176).

Nonetheless, to date, targeting biomarkers that are both prognostic and predictive is likely to improve outcome for patients with high-risk myeloma fastest but has to be demonstrated in statistically powered trials (177, 178).

## Available therapeutic strategies for refractory myeloma: focus on novel agents

Triple refractory MM is the archetype of relapsed or refractory multiple myeloma (RRMM). Data from the MAMMOTH study, which focused on CD38 mAb-refractory myeloma (179), examined resistance to several drugs and found that novel combinations of current drugs might be effective. This was the case for carfilzomib, cyclophosphamide, and Dex, which induced a 52% overall response, a median PFS of 4 months, and a median overall survival (OS) of 12 months (179). As discussed above, melflufen enables aminopeptidase activity inside MM cells but not in non-transformed cells (87). The results of the HORIZON study (88) prompted FDA approval of melflufen, based on promising results in RRMM and in triple refractory MM as well. Selinexor, an inhibitor of nuclear translocation, is another option (180), as it acts on exportin activation, which moves tumor suppressor proteins out into the cytoplasms. Vogl et al. showed that selinexor in combination with Dex was effective in both quad- and penta-refractory disease (181).

Antibody drug conjugates (ADCs) are another novel approach to RRMM management. ADCs are targeted immunotherapies composed by a cytotoxic drug fixed to an antibody scaffold directed against a tumor cell antigen. Upon binding with the tumor cell surface antigen, the ADC is internalized by the tumor cell and processed by the endo-lysosomal system leading to the release of the cancer toxic drug (182). Belantamab mafodotin is the first Food and Drug Administration (FDA)-approved BCMA targeted ADC for treatment of RRMM. It exerts a direct cytotoxic effect through the intracellular release of the monomethyl auristatin E (MMAE) and consequent spindle poisoning and apoptosis. ADCs act also *via* immunogenic cell death, by releasing, for example, HMGB1 and ATP, leading to the activation and mobilization of an immune effector response that includes T cells, macrophages, and dendritic cells. The DREAMM-2 study demonstrated the efficacy of belantamab mafodotin activity in patients with MM refractory to PIs, IMiDs, and daratumumab (183). In a longer-term follow up, PFS and OS improved in patients with a minimal response or better, but the ocular toxicities and thrombocytopenia must be addressed (184). However, due to the earlier use of novel agents, RRMM is becoming increasingly challenging to manage. The better use of currently available drugs in new combinations should thus be the goal in managing MM. Next-generation approaches should be directed at circumventing key mechanisms of resistance, while eliciting a better response with fewer toxicities. The development of new drugs with novel mechanisms of action must be supported by better predictive tools and immune-microenvironment and biology dissection (185, 186) to ensure that the right drug is delivered to the right patient at the right time (187–189).

## Author contributions

Conceptualization, AS, PL and VR. Data curation, EM, AS, MP. Writing, AS, EM, CT and PL. Supervision, PL, MC and VR. Funding, CT, MC, VR. All of the authors reviewed the manuscript, approved the draft submission, and accept responsibility for all aspects of this study. All authors have read and agreed to the published version of the manuscript.

## Funding

This work was supported by the Italian Association for Cancer Research (AIRC) through an Investigator Grant no. 20441 to VR and Investigator Grant no.22059 to MC. The sponsors of this study are non-profit organizations that support science in general; they had no role in gathering, analyzing, or interpreting the data.

## Conflict of interest

The authors declare that the research was conducted in the absence of any commercial or financial relationships that could be construed as a potential conflict of interest.

## Publisher’s note

All claims expressed in this article are solely those of the authors and do not necessarily represent those of their affiliated organizations, or those of the publisher, the editors and the reviewers. Any product that may be evaluated in this article, or claim that may be made by its manufacturer, is not guaranteed or endorsed by the publisher.
